# Barriers and facilitators of access to maternal services for women with disabilities: scoping review protocol

**DOI:** 10.1186/s13643-017-0494-7

**Published:** 2017-05-16

**Authors:** Doreen Mheta, Tivani P. Mashamba-Thompson

**Affiliations:** Discipline of Public Health Medicine, School of Nursing and Public Health, University of KwaZulu-Natal, Durban, 4001 South Africa

**Keywords:** Maternal health, Disability, Health care, Access, Sub-Saharan Africa

## Abstract

**Background:**

The Sustainable Development Goals (SDGs) emphasises the increasing equitable coverage of quality health care and provision of integrated services as means of reducing maternal mortality. Despite so much effort being placed on improved access to maternity health care, studies show that women with disabilities are being systemically excluded from the mainstream maternal health services. The proposed scoping review aims to map literature on the barriers and facilitators of access to maternal services for women with disabilities.

**Methods and analysis:**

The search strategy for this scoping review study will involve electronic databases including Pubmed, MEDLINE via EBSCOhost, CINAHL Plus with full text via EBSCOhost, Africa-Wide Info via EBSCOhost, JSTOR and Proquest Health and Medical Complete. Articles will also be searched through the “Cited by” search as well as citations included in the reference lists of included articles. A two-stage mapping strategy would be conducted. Stage 1 would be to screen studies through examining their titles. Furthermore, we will screen abstracts of the identified studies descriptively and by focus and method as stipulated by the inclusion and exclusion criteria. In stage 2, we will extract data from the included studies. A parallel screening and data extraction will be undertaken by two reviewers. We will access the quality of the included studies using the mixed methods appraisal tool (MMAT). We will use NVIVO version 10 to extract the relevant outcomes and thematic analysis of the studies.

**Discussion:**

We anticipate to find relevant studies reporting evidence on the barriers and facilitators of access to maternal health services in Sub-Saharan Africa. The evidence obtained from the included studies when summarised will help guide future research. The study findings will be disseminated electronically and in print. In addition, it will be presented at conferences related to sexual reproductive health, maternal health care and reproductive health.

## Background

Maternal health is a global health priority which emphasises the reduction of maternal mortality in developing countries. The recently adopted Sustainable Development Goals (SGDs) focuses on increasing equitable coverage of quality health care and provision of integrated services as means to reduce maternal mortality [1]. Most governments in Sub-Saharan Africa are prioritising maternal health [2, 3]. However, the majority of the countries in this region are faced with poor referral systems, shortages of skilled health personnel and poor transport infrastructure [4]. While most developed countries and some low and middle income countries (LMICs) experienced some declines in maternal mortality ratios, most of the countries in Sub-Saharan regions still experience high maternal mortality rates [5]. As a result, Sub-Saharan Africa did not achieve the millennium development goal of reducing maternal mortality by 75% [6].

South Africa is one of the developing countries that are in the forefront in the prioritising of maternal health through increasing primary health care clinics [3]. This is evidenced by the removal of user fees for maternal and child health services at the levels of primary health care and district hospital [7]. Despite these measures being put in place, women particularly the vulnerable and disadvantaged still face numerous challenges in accessing these services in South Africa [8]. While the factors that militate against maternity services for women in general are well documented in Sub-Saharan Africa, there is little research that documents the factors that inhibit or enhance access to maternity and child services for women with disabilities [9–11]. Though there are numerous definitions of disabilities, in this review, disabilities will refer to long-term visual, hearing, mental and physical impairment [8].

Access to health care is a complex phenomenon which is influenced by multiple factors. Disparities in access to health result in adverse health outcomes, thus posing a public health problem [12]. There is no single definition of access to health care services; however, a comprehensive view of access pertains to the dimensions of availability, accessibility, accommodation, affordability and acceptability [13]. For this review, access to maternal health will be explored through the availability, affordability, acceptability and quality of the services [13]. Some of the challenges that are specifically faced by women with disabilities include survival rates, maternal mortality and morbidity, accessing information on sexual reproductive health, family planning services, and prenatal and post natal services [14]. In addition, research reveals that women with disabilities have higher pregnancy complications, preterm deliveries and low birth infants [15]. Despite that there is a growing recognition that health systems should develop appropriate and accessible maternal health care services for women with disabilities [11, 14, 16], there is paucity of evidence on the experiences of women with disabilities on accessing maternal health care services [16].

The existing literature indicates that there is a need to understand the barriers and facilitators of access to maternal services as well as the different models of maternity health care services that could facilitate choices for women with disabilities [17]. Moreover, there is a need for strategies to improve access to maternal health care services for this population [14]. This may facilitate the development of horizontal approaches towards the reduction of maternal mortality in Sub-Saharan Africa. This scoping review therefore aims at mapping literature on the barriers and facilitators of access to maternal health care services for women with disabilities. The objectives of this scoping review are as follows:

- ➢ To review published literature on the barriers and facilitators of access to maternal health services for women with disabilities
- ➢ To review the literature on existing maternity health care models for women with disabilities
- ➢ To review literature on the existing interventions to improve access to maternity health care for women with disabilities

The findings from this study will enable the researchers to examine the extent and range and nature of research activity on the barriers and facilitators of access to maternity services for women with disabilities. In addition, the findings will enable the researchers to identify the different maternity health care models and interventions that improve access to maternal health care services.

## Methodology

### Scoping review

We will conduct a scoping review of peer-reviewed literature on the barriers and facilitators of access to maternal services for women with disabilities. A scoping review method was selected as it facilitates the mapping of new concepts, types of evidence and gaps related [18]. For the proposed review, we would be guided by Arksey and O’Malley framework [19]. The framework involves (i) identifying the research question, (ii) identifying relevant studies, (iii) study selection, (iv) charting the data, and (v) collating, summarising and reporting results.

#### Identifying the research question

The research question is what is known from the existing literature about the barriers and facilitators of access to maternal health services in Sub-Saharan Africa?

The sub-research questions are as follows:

1. What are the existing models of maternal health care services in Sub-Saharan Africa?
2. What are the available interventions for facilitating access to maternal services for women with disabilities in Sub-Saharan Africa?

### Eligibility of research question

The study will use an amended PICOS (Population, Intervention, Comparison, Outcomes and Study setting) framework to determine the eligibility of the research question (Table 1).

**Table 1:** PICOS framework for determination of eligibility of review question

Criteria	Determinants
Population	The population of this study will be women with disabilities (that is, visual, hearing, mental and physical impairment) who are seeking maternal health care services (antenatal, perinatal and immediate post-partum).
Intervention	Access to maternal health care services (antenatal, perinatal and immediate post-partum)
Comparison	Women without disabilities
Outcomes	Access to maternal health care services
Study setting	Sub-Saharan Africa While the review focuses mainly on studies from Sub-Saharan Africa, due to the paucity of literature on access to maternal services for women with disabilities in this region, the setting has been opened to include studies from all over the world.

#### Identifying relevant studies

Primary studies that have a clear empirical base utilising qualitative, quantitative and mixed methods published in peer-reviewed journals as well as in grey literature that address the research question will be included. All study designs would be included. An electronic search will be conducted in the following electronic databases: MEDLINE/Pubmed, CINAHL Plus with full text (EBSCO) and Africa-Wide information, Google Scholar and Proquest. Websites such as the World Health Organisation (WHO), UNICEF and governmental websites would be searched for policies and reports on access to maternal services for women with disabilities. Studies will be identified by searching literature that was published in any language and those studies that are translatable to English from January 2000 to December 2015.

Articles will also be searched through the “Cited by” search as well as citations included in the reference lists of included articles. The search terms will include maternal health, disability, health care, access, and Sub-Saharan Africa. Database-specific thesaurus terms (e.g. MeSH terms) as well as free-text terms will be used to search articles. After searching, the studies will be screened against the inclusion and exclusion criteria.

#### Study selection

The eligibility criteria were developed to ensure that the included studies contain the specific information needed to answer the research question on the barriers and facilitators of access to maternal health care services for women with disabilities.

## Eligibility criteria

### Inclusion criteria

For studies to be included, they should meet the following criteria:

- There would be no language restriction.
- Focus on women with disabilities seeking maternal health care services.
- Published from January 2000 to December 2015.
- Report on experiences of women with disabilities when accessing maternal health care services.

### Exclusion criteria

Studies will be excluded if they meet the following characteristics:

- Studies which do not have women with disabilities as part of the study population focus on women with disabilities seeking health care services other than maternal health care services: This review will exclude studies that have women without disabilities as the population of interest due to the fact that women with disabilities have their own challenges that are specific to them. Studies that report on women without disabilities may not bring out the barriers and facilitators of access to maternal services specific to women with disabilities
- Studies that report on drug and procedural interventions and
- Studies that are published before January 2000 and after December 2015: Studies conducted during the year 2016 as it is now a new era of the SDGs whereas during the years 2000 to 2015, it was the MDG era. Studies publish after December may have another focus which is completely different from the one in the MDG era. Furthermore, before 2000, the emphasis on reducing maternal deaths by three quarters did not exist and the Convention on the Rights of Persons with Disabilities (CRPD) had not been adopted by the United Nations. As result, the issues of women with disabilities were not emphasised as they were after 2006.

The search strategies will be piloted to check the appropriateness of the selected databases and key words. Articles will be searched from the databases by one reviewer who will share the Endnote library with the second reviewer. The two reviewers will conduct a comprehensive title screening guided by the eligible criteria. All eligible studies will be exported into EndNote X7.5 reference management software. EndNote X7 program will be used to check for duplication of articles and to delete the duplicated articles. Table 2 below illustrates how the electronic data search will be recorded.

**Table 2:** Electronic search record

Keyword search	Search engine used	Number of publications retrieved
ᅟ	ᅟ	ᅟ

Abstracts and full articles of the included studies will be screened for eligibility. This will be conducted independently by two reviewers to identify study analysis and assessment. Where there is no agreement between the two reviewers, the studies will be passed on to a third reviewer for consideration. We will seek for assistance from the UKZN library services for articles that are difficult to find. We will also write to the authors to ask for papers in cases of difficult to find articles. Table 4 in the appendix presents the results of the pilot search.

**Fig. 1:**
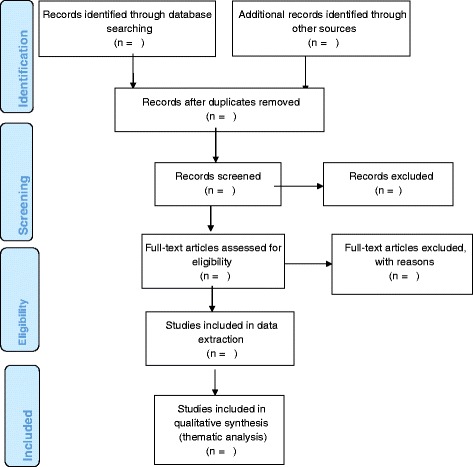
Study selection procedure

The study selection procedure will be summarised using a PRISMA chart as indicated in Fig. 1.

#### Charting the data

An analytical method would be utilised to extract the background information and process oriented information of each included study. A data charting form would be developed and piloted. The variables and themes to include in order to answer the question will be determined as indicated in Table 3. The data charting form will be constantly updated.

**Table 3:** Data charting form

Author and date	ᅟ
Study title	ᅟ
Journal full reference	ᅟ
Aims or research question	ᅟ
Participant characteristics	ᅟ
Recruitment context (e.g. where people were recruited).	ᅟ
Sampling method	ᅟ
Study design	ᅟ
Theoretical background	ᅟ
Data collection (what data collection methods were used?)	ᅟ
Data analysis (how was the data analysed?)	ᅟ
Intervention	ᅟ
Intervention outcome	ᅟ
Most relevant findings	ᅟ
Conclusions	ᅟ
Comments	ᅟ

#### Collating, summarising and reporting of results

The aim of this study is to map the existing evidence and to summarise the findings as presented across articles. A narrative account of the data extracted from the included studies will be analysed using the thematic content analysis. Data will be extracted around the following outcomes: models of maternity health care services, barriers of access to maternity health care services, and facilitators of access to maternal health care services. Emerging themes will also be coded. NVIVO software version 10 would be utilised collectively to code the data from the included studies based on the above categories [20]. The below process would be followed;

- Coding data from the included articles
- Categorising the codes into major themes
- Displaying the data
- Identifying key patterns in the data and identify sub-themes
- Summarising

### Synthesis

We will analyse the resulting themes and critically examine their relationship to the research question. The reviewers will also analyse the meanings of the findings in relation to the aim of the study and the implications of these findings for future research, policy and practice.

### Quality appraisal

The quality of the studies will be determined through study appraisal using the mixed method appraisal tool (MMAT)-Version 2011 [21]. The tool will be utilised to examine the appropriateness of the aim of the study, adequacy and methodology, study design, participant recruitment, data collection, data analysis, presentation of findings, authors’ discussions and conclusions. The quality of the article will be determined from the examination of the above mentioned aspects.

## Discussion

The scoping review will be conducted as a first part of the study on the barriers and facilitators of access to maternity services for women with disabilities in South Africa. The review is aimed mapping the existing evidence and summarising the findings as presented across the studies on the barriers and facilitators of access to maternal health care services for women with disabilities. In addition, the review will on identify the existing maternity models and interventions that enhance access to maternal health care services for women with disabilities. Despite that there is a growing recognition that health systems should develop appropriate and accessible maternal health care services for women with disabilities [11, 14, 16], there is paucity of evidence on the experiences of women with disabilities on accessing maternal health care services [16]. In order to enable development of disability friendly maternal health care services, there is a need to explore the maternal health care needs, barriers and facilitators of access to maternal services for women with disabilities especially in low and middle income countries [15].

Studies that report on drugs and procedural interventions would be excluded because focus of this review is on access to maternal health services. Most maternal deaths could be avoided if the quality maternal health care services are available to those who need the services. Therefore, this review excludes studies that report on drug and procedural interventions as the main focus is on access (availability, affordability, acceptability and quality of the services). The studies on drug and procedural interventions report on women with disabilities who have already accessed the services and the intervention is not the focus of this study.

The findings of this study may be of interest to policy makers and stakeholders involved in the provision of maternal health care services, and stakeholders advocating for equity of access and health systems strengthening. In addition, the findings of this study will be of interest to researchers by highlighting gaps in evidence that may require further investigation.

## Appendix

**Table 4:** Results of the pilot database search

Keyword search	Date of search	Search engine used	Number of publications retrieved
(((“disabled persons”[MeSH Terms] OR (“disabled”[All Fields] AND “persons”[All Fields]) OR “disabled persons”[All Fields] OR “disabled”[All Fields]) AND (“women”[MeSH Terms] OR “women”[All Fields])) AND (access[All Fields] AND (“maternal health services”[MeSH Terms] OR (“maternal”[All Fields] AND “health”[All Fields] AND “services”[All Fields]) OR “maternal health services”[All Fields] OR (“maternal”[All Fields] AND “health”[All Fields] AND “care”[All Fields]) OR “maternal health care”[All Fields]) AND services[All Fields])) OR (models[All Fields] AND (“maternal health services”[MeSH Terms] OR (“maternal”[All Fields] AND “health”[All Fields] AND “services”[All Fields]) OR “maternal health services”[All Fields])) AND ((“loattrfull text”[sb] AND hasabstract[text] AND “loattrfree full text”[sb]) AND (“2000/01/01”[PDAT] : “2015/12/31”[PDAT]) AND “humans”[MeSH Terms])	11 April 2017	MEDLINE via Pubmed	1115

## Abbreviations

CRPD: Convention on the Rights of Persons with Disabilities
LMIC: Low and middle income countries.
MDG: Millennium development goals
MMAT: Mixed methods appraisal tool
PICOS: Population intervention outcomes study setting
SDG: Sustainable Development Goals
